# Benchmarking of Multispectral Pansharpening: Reproducibility, Assessment, and Meta-Analysis

**DOI:** 10.3390/jimaging11010001

**Published:** 2024-12-24

**Authors:** Luciano Alparone, Andrea Garzelli

**Affiliations:** 1 Department of Information Engineering, University of Florence, 50139 Florence, Italy; 2 Department of Information Engineering and Mathematics, University of Siena, 53100 Siena, Italy; andrea.garzelli@unisi.it

**Keywords:** benchmarking, haze correction, meta-analysis, pansharpening, remote sensing, reproducibility

## Abstract

The term pansharpening denotes the process by which the geometric resolution of a multiband image is increased by means of a co-registered broadband panchromatic observation of the same scene having greater spatial resolution. Over time, the benchmarking of pansharpening methods has revealed itself to be more challenging than the development of new methods. Their recent proliferation in the literature is mostly due to the lack of a standardized assessment. In this paper, we draw guidelines for correct and fair comparative evaluation of pansharpening methods, focusing on the reproducibility of results and resorting to concepts of meta-analysis. As a major outcome of this study, an improved version of the additive wavelet luminance proportional (AWLP) pansharpening algorithm offers all of the favorable characteristics of an ideal benchmark, namely, performance, speed, absence of adjustable running parameters, reproducibility of results with varying datasets and landscapes, and automatic correction of the path radiance term introduced by the atmosphere. The proposed benchmarking protocol employs the haze-corrected AWLP-H and exploits meta-analysis for cross-comparisons among different experiments. After assessment on five different datasets, it was found to provide reliable and consistent results in ranking different fusion methods.

## 1. Introduction

Several decades ago, orbiting satellites for Earth observation (EO) started collecting considerable amounts of data of crucial relevance to human activities. The ever increasing availability of satellite images of Earth, like (i) optical data featuring spectral diversity (visible–near infrared (VNIR) and short-wave infrared (SWIR)) with complementary spectral and spatial resolutions, (ii) thermal infrared (TIR) data, and (iii) microwave data from X-, C-, and L-band synthetic aperture radar (SAR), possibly with multipolarization capability, together with the peculiar characteristics of different imaging modalities, have fostered the study of fusion methods. The purpose of fusion is to produce an additional value, in addition to those available separately from each data set [1]. These data are used both as intermediate products and for visual interpretation. Although the results of fusion may be interpreted by humans, partially supervised and fully automated systems, most notably thematic classifiers, may take advantage of the products of fusion instead of relying on each dataset alone in solving specific detection and monitoring tasks, e.g., landslides, floods, droughts, and fires.

Extensive research on the fusion of RS images for EO has been carried out over the last several decades, and a considerable number of fusion methods have been developed. Image fusion can be classified following several perspectives. One is based on the homogeneity of the sensors; homogeneous data fusion merges images that are captured by instruments exploiting the same imaging mechanism. This category is regarded as the fusion of unimodal images. The fusion of multispectral (MS) and panchromatic (Pan) data is referred to as MS pansharpening and is perhaps the most typical example of unimodal fusion. The data sets subject to unimodal fusion are the result of measurements of reflected solar radiation in different wavelengths and with different spatial resolutions. Another approach involving the fusion of heterogeneous data, i.e., multimodal fusion, concerns all cases where the data sets are captured by instruments that do not share the same imaging modes.

An additional way to discriminate fusion methods lies in the content level that is subject to fusion: (i) the pixel level (or area-based approaches), (ii) the feature level, and (iii) the decision level [1]. The fusion at the pixel level combines the values of pixels in the merged images; the goal is to produce a fused image pixel by pixel. Feature-level fusion incorporates specific descriptors (features) extracted separately from the images to be merged. For the fusion of optical and SAR images, direct pixel-level combination of the data is not recommended, to avoid contamination of the fused image with the low signal-to-noise ratio (SNR) typical of SAR data [2]. Instead, fusion is performed through features such as texture and spatial heterogeneity calculated from SAR images [3]. These can be implanted in the optical images, thereby relaxing the tight co-registration required by pixel-level fusion. Finally, decision-level fusion merges classification maps obtained separately from each dataset or produced by different classifiers on the same dataset. Thus, the output of decision-based fusion is a classification map [4].

Concerning optical data, or better yet, data captured in the reflective part of the spectrum, the growing demand of products with ever increasing resolution is pushing toward the design of acquisition devices featuring high performance. However, because of intrinsic constraints on the number of photons per pixel of each band and the electronic noise, which together determine the signal-to-noise ratio (SNR) [5], very fine spatial and spectral resolutions cannot be achieved by unique instruments. Image fusion represents a viable solution to overcome this issue by combining broad-spectrum images with high spatial resolution and multichannel data with lower spatial resolution [1,6]. Among area-based methods, MS pansharpening is an established methodology which has received considerable attention [1,7] since the advent of very/extremely high resolution (V/EHR) satellite MS scanners. In synthesizing a unique product featuring the spectral bands of the original MS image at the spatial resolution of Pan, pansharpening benefits from the complementary spatial and spectral resolutions of MS and Pan due to physical constraints in the SNR of the broad and narrow bands [8]. However, it is important to highlight that pansharpening cannot increase the spatial resolution of the spectral information of the original data unless suitable prior models of the underlying scene are devised. Instead, it is a way to represent such information at a finer scale that is more suitable for visual or automated analysis tasks [1,7,9].

Though the specific term was first introduced in 1996, pansharpening dates back to the launch of the first SPOT satellite (Satellite pour l’Observation de la Terre), the first spaceborne system equipped with a panchromatic instrument, on 22 February 1986. A first generation of fusion methods rapidly flourished around the first generation of SPOT, 1, 2, and 3, lasting into 1996. The common feature of all such methods is that the quality is measured visually or by empirical criteria [10] often borrowed from computer vision. Note that no evidence has ever been provided to indicate that these criteria are suitable for remote sensing. These landmark methods survived over time, and served as specimens for use in more advanced and performant techniques. Other methods, though very popular at times, similarly disappeared within a few years when outperformed by newer conceptions of the problem. A notable case is fusion based on principal component analysis (PCA), which disappeared after the introduction of Gram–Schmidt (GS) spectral sharpening [11]. In fact, it was later recognized that GS is a generalization of PCA [1]. Methods based on extension of the intensity hue saturation (IHS) color space to an arbitrary number of bands have also been progressively abandoned, although they have been rediscovered in recent years [12,13].

**Figure 1:**
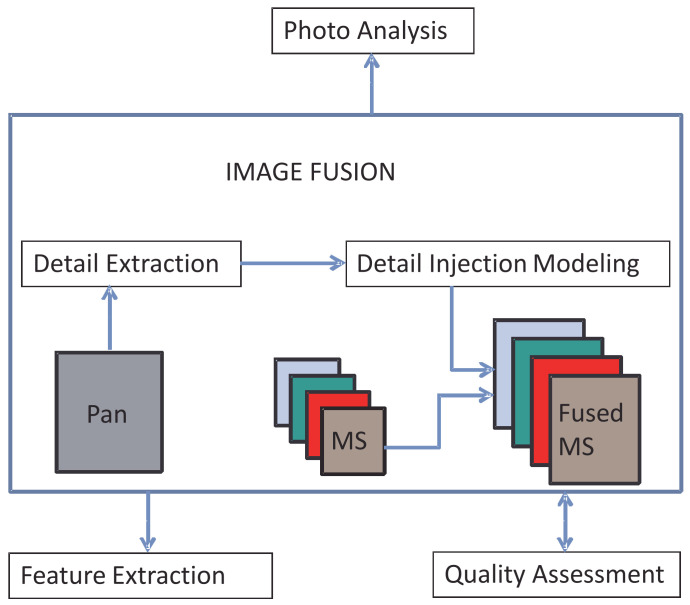
General flowchart of MS pansharpening and related application scenario. The colors of the MS bands loosely indicate blue, green, red, and NIR.

The second generation of pansharpening methods, which started in 1997 after the landmark article on quality assessment by [14] and was established several years later by [9], features several methods following the same flowchart. After the MS bands have been interpolated and overlapped with the Pan image [15], the co-registration between the two datasets is first checked [16,17]. Spatial details are then extracted from Pan and added to the resampled MS bands according to the injection model, which can be based on any criteria, for example, genetic algorithms [18] or interscale predictions achieved by Kalman filtering [19]. The stage of detail extraction can follow the spectral approach, originally known as component substitution (CS), or the spatial approach, relying on separable [20,21] or nonseparable [22] multiresolution analysis (MRA). Formal MRA was quickly abandoned after its intrinsic drawbacks were recognized [23]. According to the spectral criterion, the spatial detail is provided as the difference between the original Pan image and an intensity component, achieved by the pixel combination of the interpolated MS bands. Following the spatial criterion, the detail is calculated as the difference between the original Pan and its lowpass-filtered version, which retains the same spatial frequency content as the MS bands [24]. The two classes achieve fused products with complementary features, such as spectral/spatial distortions and robustness to spatial/temporal misalignment [25]. Better yet, spectral and spatial methods behave complementarily with respect to their tolerance to spatial and spectral defects, respectively; whereas the former consist of aliasing and misregistration [26], the latter occur when the two datasets are captured from different platforms and/or on different dates [27]. This feature is invaluable for the fusion of multi-platform data [28,29]. Note that the intensity component does not make sense for multimodal fusion, if it can even be defined [30]. Figure 1 outlines the MS pansharpening procedure and highlights possible application scenarios.

The Pan image is preliminarily histogram matched, that is, radiometrically transformed by constant gain and offset in such a way that its lowpass version with the same spatial frequency content as the MS bands exhibits mean and variance equal to those of the spectral component to be replaced (the intensity component for CS methods or the MS band to be sharpened for MRA methods [31]). Models of detail injection govern the combination of the spatial details of Pan with the resampled MS bands. This model is stated between each band and the lowpass-filtered Pan image. The multiplicative injection model with haze correction [32,33] is capable of improving fusion, by exploiting imaging mechanisms through the atmosphere [34]. The design of suitable injection models is a key topic in multimodal fusion, where data are the result of different physical mechanisms such as thermal sharpening [35]. Furthermore, any baseline pansharpening method, CS or MRA, can be specialized to take advantage of a potentially space-varying context [36,37] through proper injection modeling. Though often indicated as a third class, hybrid methods are actually a cascade of methods from the two classes of CS and MRA. The most notable approach in this category is the additive wavelet luminance proportional (AWLP) method [38]. The AWLP method proportionally injects the spatial details of Pan into the modulus of the MS pixel vector. Spatial details are extracted from Pan through a prototype filter derived from the choice of $B_{3}$ cubic-spline scaling function [38]. The literature shows that AWLP is capable of attaining state-of-the-art performance [9,39].

The baseline categorization into CS and MRA has recently been upgraded by the introduction of other methods [7] relying on Bayesian inference [40], total variation (TV) regularization [41], and super-resolution [42,43,44]. In particular, the firt pioneering achievements in MS pansharpening that exploited the concept of super-resolution [45,46,47] appeared in the literature more than a decade ago. Despite the formal mathematical elegance of some of these approaches, the best methods exhibited only a very subtle increase in performance over the state-of-the-art [48,49], often obtained at an exorbitant computational cost involving massive constrained numerical minimization with plenty of adjustable parameters, including those governing the prior model of the imaged surface and the convergence of the solution. Methods based on super-resolution, or more generally on optimization-based variational methods, either model-based [50,51] or not [52,53,54], are inconceivable for practical applications requiring routine fusion of tens of megapixels of data, for which traditional approaches are pursued. Within the past decade, machine learning paradigms have been rediscovered for fusion, from the pioneering approach relying on convolutional neural networks (CNN) [55] to sophisticated architectures such as generative adversarial networks (GAN) [53]. For learning-based methods, the stages of histogram matching and detail injection modeling are automatically learned from the training data and implicitly performed when the network is run; on the other hand, GANs are able to control each other, and as such are invaluable for multimodal fusion, among other approaches [56]. Here, we wish to stress a concept that is developed in the rest of this paper, namely, new-generation methods relying on variational optimization (VO). These can be based on either modeling or learning; however, despite their high performance in certain cases, they may be unsuitable for benchmarking in direct comparisons, that is, with optimization of benchmarks on the dataset of each experiment. In fact, their performance is crucial, being subordinated on one side to proper optimization of the running parameters on a local basis, e.g., on small blocks partially overlapped to avoid discontinuities in fusion effects, and on the other to proper selection of training data.

This paper investigates how to assess and rank pansharpening methods, rather than pansharpened data products; in other words, how to make comparisons that are as independent as possible of the specific dataset, e.g., instrument and landscape. The proposed benchmarking protocol is stated for a reduced resolution assessment, but may also be extended to full-scale evaluations. It specifies the experimental setup, e.g., data format, degradation, and interpolation procedures, most suitable quality/distortion indexes, and most suitable benchmarks, that is, the methods that are used for comparisons from which conclusions are drawn. In parallel, the ideal benchmark is investigated. Desirable features are high performance, fast algorithms, lack of parametric adjustments, and reproducibility of results. Focusing on haze correction for hybrid methods, specifically AWLP, we first recall some issues in [33] that provide an interpretation of multiplicative injection models in terms of the radiative transfer model governing the acquisition of an MS image from a real-world scene. Afterwards, we show the condition under which the AWLP method collapses into a multiplicative method, for which haze correction is beneficial [57], and derive the correction. The haze-corrected AWLP, called AWLP-H [58], is assessed on five real datasets acquired by IKONOS, QuickBird, WorldView-2, WorldView-3, and GeoEye-1 over different landscapes. Assessment is performed at reduced resolution following Wald’s protocol [8,14]. For all test cases, the best performance is achieved against the state-of-the-art CS and MRA methods taken from [9]. The improvement in performance with respect to the baseline AWLP is particularly clear when vegetated scenarios are concerned.

The remainder of this paper is organized as follows. Section 2 reviews CS, MRA, and hybrid methods, with a focus on AWLP; an improved version of AWLP featuring haze correction is proposed as an ideal benchmark to evaluate new and existing pansharpening methods, and the characteristics of a fair and standardized comparison of pansharpening methods are illustrated with a focus on reproducibility of results. Section 3 is devoted to the experimental results and comparisons, achieved through a meta-analysis carried out on five different datasets. A discussion of the results is presented in Section 4. Finally, our conclusions are drawn in Section 5.

## 2. Materials and Methods

Existing pansharpening methods up to the second generation can be labeled as either CS or MRA based on the unique difference in the way details are extracted from Pan, which has an impact on the fused images [9,26]. Hybrid methods include CS followed by MRA and MRA followed by CS [31], and as such are equivalent to either CS-based or MRA-based methods. The injection model is inherited from the dual-class method. First, the notation used in this study is presented; then, a survey of CS and MRA serves as an introduction to hybrid methods, focusing on AWLP-H and haze correction.

### 2.1. Notation

The math notation is defined as follows. Vectors are indicated in bold lowercase, e.g., x, with the *i*th element defined as $x_{i}$, while 2D and 3D arrays are denoted in bold uppercase, e.g., X. An MS image $M={\left\{M_{k}\right\}}_{k=1,\ldots ,N}$ is a 3D array composed of *N* spectral bands, each being a 2D array, indexed by the subscript *k*. Hence, $M_{k}$ represents the *k*th spectral band of M. The Pan image is a 2D array, and is indicated as P. The interpolated and pansharpened *k*th band of the MS image are denoted as ${\tilde{M}}_{k}$ and ${\hat{M}}_{k}$, respectively. Unlike the conventional matrix product and ratio, such operations are intended to represent the product and ratio of terms having the same positions within the array.

### 2.2. Component Substitution Methods

The class of spectral (or CS) methods is based on the projection of the MS image into another vector space by assuming that the forward transformation splits the spatial structure and spectral diversity into separate components. Here, the term ‘spectral’ means that spatial details are extracted by processing the data cube constituted by the stack of bands only along the *z* axis, that is, in the spectral direction.

Under the hypothesis of substitution of a single component that is a linear combination of the input bands, the fusion process can be obtained without explicit calculation of forward and backward transformations, instead following a proper injection scheme [1]. This results in fast implementation of CS methods, for which the general formulation is as follows:

(1) $${\hat{M}}_{k}={\tilde{M}}_{k}+G_{k}\;\cdot \;\left({\bar{P}}^{\left(I_{L}\right)}-I_{L}\right),\;\;\;\;\;k=1,\ldots ,N$$

in which *k* is the band index, $G=[G_{1},\ldots ,G_{k},\ldots ,G_{N}]$ is the 3D array of injection gains, which in principle may be one per pixel per band, and the intensity $I_{L}$ is defined as

(2) $$I_{L}=w_{0}+\overset{N}{\sum_{i=1}}w_{i}\;\cdot \;{\tilde{M}}_{i},$$

where the vector $w=[w_{1},\ldots ,w_{i},\ldots ,w_{N}]$ contains the spectral weights and is equal to the first row of the matrix that defines the forward transform. The term ${\bar{P}}^{\left(I_{L}\right)}$ is the result of the histogram matching of P to $I_{L}$:

(3) $${\bar{P}}^{\left(I_{L}\right)}≜\left(P-\mu_{P}\right)\;\cdot \;\frac{\sigma_{I_{L}}}{\sigma_{P_{L}}}+\mu_{I_{L}}$$

in which μ and σ denote the mean and square root of the variance, respectively, and $P_{L}$ is a lowpass version of P having the same spatial frequency content as $I_{L}$ [31].

In Gram–Schmidt (GS) spectral sharpening [11], the fusion process is described by Equation (1), where the injection gains are constant for each band, and as such are denoted as ${\left\{g_{k}\right\}}_{k=1,\ldots ,N}$. They are provided by [59]

(4) $$G_{k}=g_{k}=\frac{\mathrm{cov}({\tilde{M}}_{k},I_{L})}{\mathrm{var}(I_{L})}\;\;\;\;\;k=1,\ldots ,N,$$

in which $\mathrm{cov}\left(X,Y\right)$ indicates the covariance between X and Y, with $\mathrm{var}\left(X\right)$ as the variance of X. In [59], a multivariate linear regression was exploited to model the relationship between the lowpass-filtered Pan $P_{L}$ and interpolated MS bands:

(5) $$P_{L}={\hat{w}}_{0}+\sum_{i=1}^{N}{\hat{w}}_{i}\;\cdot \;{\tilde{M}}_{i}+\epsilon ≜{\hat{I}}_{L}+\epsilon$$

where ϵ denotes the space-varying residue and ${\hat{I}}_{L}$ is the intensity component. The set of weights ${\left\{{\hat{w}}_{k}\right\}}_{k=1,\ldots ,N}$ is the minimum-MSE (MMSE) solution of Equation (5). A measure of the success of the match achieved by Equation (5) is the coefficient of determination (CD), or $R^{2}$, provided by

(6) $$R^{2}≜1-\frac{\sigma_{\epsilon }^{2}}{\sigma_{P_{L}}^{2}},$$

where $\sigma_{\epsilon }^{2}$ represents the variances of the residue ϵ with zero mean and $\sigma_{P_{L}}^{2}$ represents the residue of the lowpass-filtered Pan. The histogram matching of Pan to ${\hat{I}}_{L}$ accounts for $\mu_{P}=\mu_{P_{L}}=\mu_{{\hat{I}}_{L}}$ from Equation (5); hence, from the definition of CD stated in Equation (6), we have

(7) $${\bar{P}}^{\left({\hat{I}}_{L}\right)}=\left(P-\mu_{P}\right)\;\cdot \;R+\mu_{P}.$$

Multiplicative or contrast-based injection gain is a particular case of Equation (1) in which G is defined such that

(8) $$G_{k}=\frac{{\tilde{M}}_{k}}{I_{L}},\;\;\;\;\;\;k=1,\ldots ,N.$$

The outcome is a pansharpening method described by

(9) $${\hat{M}}_{k}={\tilde{M}}_{k}+\frac{{\tilde{M}}_{k}}{I_{L}}\;\cdot \;\left({\bar{P}}^{\left(I_{L}\right)}-I_{L}\right)={\tilde{M}}_{k}\;\cdot \;\frac{{\bar{P}}^{\left(I_{L}\right)}}{I_{L}},\;\;\;\;\;k=1,\ldots ,N,$$

where the case of all spectral weights equal to 1/N yields the popular Brovey transform (BT) method [60]. The multiplicative injection model is suitable for merging heterogeneous datasets, such as optical and synthetic aperture radar (SAR) data [61].

### 2.3. Multiresolution Analysis Methods

The spatial approach, originally referred to as MRA-based approach, relies on the injecting the high-pass spatial details of the Pan image into the resampled MS bands. According to the newer understanding of fusion based on MRA [24], it uniquely depends on a lowpass filter that generates the baseband component of the Pan image. Thus, with maximum generality, MRA-based fusion may be stated as follows:

(10) $${\hat{M}}_{k}={\tilde{M}}_{k}+G_{k}\;\cdot \;\left({\bar{P}}^{\left({\tilde{M}}_{k}\right)}-{\bar{P}}_{L}^{\left({\tilde{M}}_{k}\right)}\right),\;\;\;\;\;\;k=1,\ldots ,N$$

where Pan is histogram-matched with the resampled *k*th MS band [31]:

(11) $${\bar{P}}^{\left({\tilde{M}}_{k}\right)}≜\left(P-\mu_{P}\right)\;\cdot \;\frac{\sigma_{{\tilde{M}}_{k}}}{\sigma_{P_{L}}}+\mu_{{\tilde{M}}_{k}}$$

and ${\bar{P}}_{L}^{\left({\tilde{M}}_{k}\right)}$ is the lowpass-filtered version of ${\bar{P}}^{\left({\tilde{M}}_{k}\right)}$. According to Equations (3) and (11), histogram matching of P always implies calculating its lowpass version $P_{L}$.

Equation (10) states that the different approaches and methods belonging to this class are uniquely characterized by the lowpass filter used to obtain the image $P_{L}$, the presence or absence of a decimator/interpolator pair [21,24,62] used to achieve a Laplacian pyramid or atrous decomposition, respectively, and by the injection gains, which can be either constant ${\left\{g_{k}\right\}}_{k=1,\ldots ,N}$ or space-varying ${\left\{G_{k}\right\}}_{k=1,\ldots ,N}$.

The detail-injection model of GS spectral sharpening reported in Equation (4), referred to as projection-based gain, can also be used in conjunction with spatial methods. In this case, Equation (4) becomes

(12) $$G_{k}=g_{k}=\frac{\mathrm{cov}({\tilde{M}}_{k},P_{L})}{\mathrm{var}(P_{L})}\;\;\;\;\;k=1,\ldots ,N.$$

Per the definitions of variance and covariance, preliminary histogram matching of Pan is not required thanks to the ratio in Equation (12).

The contrast-based version of MRA pansharpening is

(13) $$\begin{array}{ccc} {\hat{M}}_{k} & = & {\tilde{M}}_{k}+\frac{{\tilde{M}}_{k}}{{\bar{P}}_{L}^{\left({\tilde{M}}_{k}\right)}}\;\cdot \;\left({\bar{P}}^{\left({\tilde{M}}_{k}\right)}-{\bar{P}}_{L}^{\left({\tilde{M}}_{k}\right)}\right)\\ & = & {\tilde{M}}_{k}\;\cdot \;\frac{{\bar{P}}^{\left({\tilde{M}}_{k}\right)}}{{\bar{P}}_{L}^{\left({\tilde{M}}_{k}\right)}},\;\;\;\;\;\;\;\;\;\;\;\;\;\;\;\;\;\;\;\;\;\;k=1,\ldots ,N. \end{array}$$

Note that unlike Equation (9), Equation (13) does not retain the spectral angle, as the modulating term depends on *k*.

**Figure 2:**
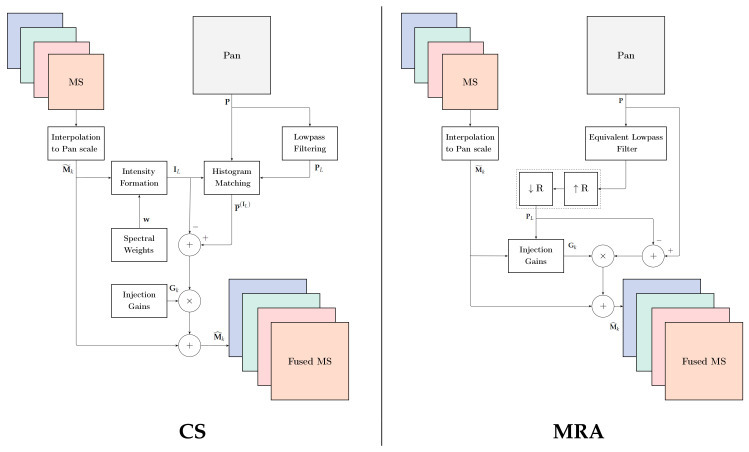
Flowcharts of the CS and MRA pansharpening methods. The ratio of the scales of MS and Pan is denoted by *R*.

Equation (13) accommodates high-pass modulation (HPM) [63] and smoothing filter-based intensity modulation (SFIM) [64], which differ from one another in the lowpass filter used to achieve $P_{L}$. Figure 2 outlines the respective flowcharts of CS and MRA pansharpening.

### 2.4. Hybrid Methods

In some cases, the spectral transformation of CS methods is cascaded with MRA to extract the injected spatial details. The resulting methods are called hybrid methods [65,66]. The most popular hybrid method with a multiplicative injection model is AWLP [38]:

(14) $${\hat{M}}_{k}={\tilde{M}}_{k}+\frac{{\tilde{M}}_{k}}{I_{L}}\;\cdot \;\left({\bar{P}}^{\left(I_{L}\right)}-{\bar{P}}_{L}^{\left(I_{L}\right)}\right)\;\;\;\;\;k=1,\ldots ,N$$

where the lowpass filter is a 5 × 5-separable $B_{3}$ spline kernel. It is noteworthy that Equation (14) cannot be written as a product, unlike Equations (9) and (13).

According to the recent categorization of pansharpening methods [1,24], hybrid methods are equivalent to either spectral or spatial methods depending on whether the injected detail is ${\bar{P}}^{\left(I_{L}\right)}-I_{L}$ or ${\bar{P}}^{\left({\tilde{M}}_{k}\right)}-{\bar{P}}_{L}^{\left({\tilde{M}}_{k}\right)}$. Thus, AWLP is a spatial method, i.e., MRA, and its histogram matching should be provided by Equation (11) instead of Equation (3).

Histogram matching was corrected in [31], and was found to be beneficial for performance:

(15) $${\hat{M}}_{k}={\tilde{M}}_{k}+\frac{{\tilde{M}}_{k}}{I_{L}}\;\cdot \;\left({\bar{P}}^{\left({\tilde{M}}_{k}\right)}-{\bar{P}}_{L}^{\left({\tilde{M}}_{k}\right)}\right)\;\;\;\;\;k=1,\ldots ,N.$$

The improved AWLP described in Equation (15) has been further enhanced by means of dehazing, just as for CS and MRA methods [33,57]. In addition, the MMSE intensity component ${\hat{I}}_{L}$ defined in Equation (5) has been used instead of $I_{L}$ in Equation (2), with ${\left\{w_{i}=1/N\right\}}_{i=1,\ldots ,N}$, as in the original publication [38].

This correction was derived by Lolli et al. [33] from their consideration of the multiplicative model in terms of radiative transfer [34], which governs the acquisition of Earth’s surface illuminated by sunlight passing through the atmosphere. We remark here that in order to have ${\hat{I}}_{L}$ close to $P_{L}$, $\hat{w}$ should be estimated through linear multivariate regression [59]. Thus, we can obtain the dehazed AWLP [58] starting from Equation (15):

(16) $${\hat{M}}_{k}={\tilde{M}}_{k}+\frac{{\tilde{M}}_{k}-H_{k}}{{\hat{I}}_{L}-H_{{\hat{I}}_{L}}}\;\cdot \;\left({\bar{P}}^{\left({\tilde{M}}_{k}\right)}-{\bar{P}}_{L}^{\left({\tilde{M}}_{k}\right)}\right)$$

where $H_{k}$ indicates the haze term of the *k*th MS band, ${\hat{I}}_{L}$ is the MMSE intensity obtained by Equation (5), and $H_{P}=H_{P_{L}}=H_{{\hat{I}}_{L}}$ is the haze term of both the intensity and Pan, either filtered by lowpass or not:

(17) $$H_{P}=H_{P_{L}}=H_{{\hat{I}}_{L}}={\hat{w}}_{0}+\overset{N}{\sum_{k=1}}{\hat{w}}_{k}\;\cdot \;H_{k}.$$

The dark object subtraction method [67] may be used to estimate the atmospheric path radiance (i.e., haze) of each channel. By assuming that at least one dark pixel having zero reflectance exists within a band, the spectral radiance of such a pixel is due to the atmospheric path radiance [67], which is assumed to be uniform over the scene. Hence, the coefficient $H_{k}$ is estimated as the minimum of the *k*th MS band [32]. Equation (16) does not require any parametric adjustment to yield the haze values in Equation (17).

### 2.5. Assessment

Evaluating the quality of pansharpened images has been the subject of extensive studies for almost three decades [14]. The quality has been observed to change with the absolute scale at which fusion is performed, not only with the relative scale of MS to Pan [20,68,69]. In fact, quality is crucial for instruments that use the blue channel and Pan’s bandwidth comprising the red edge and part of the NIR wavelengths [1], as the NIR channel is uncorrelated with the visible channels in the presence of vegetation [70]. This occurs because LandSat 7 ETM+ has been equipped with a Pan band.

**Figure 3:**
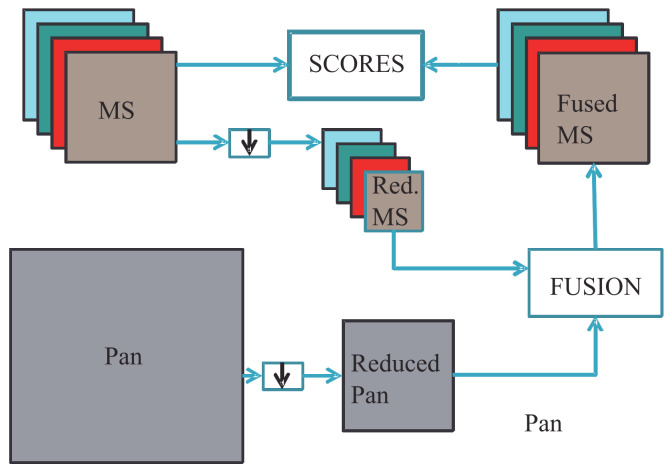
Flowchart of reduced-resolution quality assessment. The symbol ↓ denotes downsampling achieved through lowpass filtering and decimation by the MS-to-Pan scale ratio.

Following publication of the seminal paper by L. Wald [14] in 1997, it has been recognized that quality evaluation plays a major role in the definition and development of MS pansharpening methods. The original protocol foresees two separate checks:

1. Consistency, checked at the spatial scale of the fusion product.
2. Synthesis, checked at a spatial scale that is *r* times greater than that of the original Pan (with *r* in the MS-to-Pan scale ratio), as outlined in Figure 3.

In past studies, usually only the synthesis property has been checked [39]. More recently, it has been recognized that consistency also plays a key role [71]. The main advantage of synthesis, which involves a reduced-resolution (RR) test, is the accuracy of the performance assessment based on the presence of a reference image (ground truth (GT) equal to the original MS) and the availability of reliable and nonparametric similarity/dissimilarity measures [72], the results of which cannot be changed by adjusting running parameters. The main drawbacks are related to the implicit assumption of the scene’s scale-invariance. In addition, the bias introduced into the simulation procedure by the use of spatial filters, both for spatial degradation of the datasets in Figure 3 and for filtering Pan to obtain $P_{L}$, will likely favor MRA rather than CS [9]. This is because the unknown MTF (modulation transfer function) [73], that is, the spatial frequency response of the imaging system, becomes identical to the frequency response of the separable 2D filter used for spatial degradation.

These disadvantages can be overcome by resorting to a full-resolution (FR) assessment; we recall here that the synthesis property in Figure 3 is not applicable to multimodal fusion [30], while consistency always holds. However, the consistency of Wald’s protocol is a necessary but not sufficient condition unless coupled with the synthesis property [14,71]. As Wald’s consistency is spectral, an analogous spatial consistency measure was introduced later by Zhou et al. [74] to achieve a condition that is both necessary and sufficient. Unfortunately, Zhou’s protocol presumably ignored Wald’s protocol, and as a result the proposed spectral consistency was flawed. However, in conceiving a counterpart in terms of spatial consistency, they laid the foundations for the full-scale evaluations in [75,76].

The inconvenience of FR assessment represents a reduction in the significance of evaluations, as the quality is not directly measured based on the similarity to the GT but is instead either inferred from indirect consistency measurements [71,74,75,76,77,78,79] or extrapolated from reduced resolution measurements [80,81]. Contemporary studies [82] have suggested that the original MS image may not be suitable for fusion, especially for consistency measurements, due to the presence of uncorrected local shifts caused by uncompensated parallaxes originating from different viewpoints along the orbit. Inconsistencies in FR protocols have previously been noted [79,83], although the reasons were unclear. In the present context, the term quality represents the fidelity to a hypothetically available reference, and has no relationship with the intrinsic quality of the data produced by the instrument.

Considering the objective of this work, the assessment at reduced resolution is more reliable and expedites meta-analysis based on other publications [83]. Thus, the availability of a GT allows for profitable utilization of reliable and widespread vector similarity indexes. In this work, we use: (i) the ERGAS [9,39,84] index (French acronym for relative dimensionless global error in synthesis), that is, the cumulative multiband extension of the normalized root mean square error (NRMSE); (ii) spectral angle mapping (SAM) [9,85], which denotes the spatial average of the absolute value of the spectral angle between the fused and original pixel vectors, usually expressed in degrees; and (iii) the Q4/Q8 [86,87] index, which is the vector extension (using hypercomplex algebra) of the universal image quality index (UIQI) [88], which is suitable for jointly measuring both radiometric and spectral distortions of images having (up to) 4/8/$2^{n}$ bands [89]. The index is inspired by the normalized interferogram, widely used to extract phase measures from a pair of overlapped (complex) coherent images [90]. Accordingly, the MS pixel is regarded as a (hyper)complex number having as many components as the spectral bands. The meaning of the multivariate (hyper)phase has never been investigated thus far in application contexts. Although rarely used outside the scope of pansharpening, Q$2^{n}$ can be used to check the similarity of RGB color images, leaving an empty component (any one) of quaternions (hypercomplex numbers with four components). As commonly recommended, we wish to remark that all the indexes used as quality measurement methods in the present study are nonparametric.

### 2.6. Reproducibility

The term reproducibility, referring to the reproducibility of results, has multiple facets. A computational method for remote sensing data is said to be reproducible if the fusion performance is unaffected when the type of satellite data varies (e.g., sensor type, resolution, land cover); in other words, the ranking of reproducible methods does not change when carried out on data from different satellites, at different spatial scales, or with different landscapes/land cover types. Similarly, we can speak of reproducibility towards formats when the performance does not depend on the physical format of the data, e.g., spectral radiance or surface reflectance, or on the computer format, such as the use of fixed points for storage and transmission or floating points for processing and display. Notably, quality measures can also be influenced by the data format [89]. Another type of reproducibility concerns the results of processing when user experience varies; processing methods that have adjustable parameters are inevitably prone to this inconvenience.

Remote sensing image data are generally distributed in fixed-point formats of 8 to 16 bits per pixel per spectral component, together with a series of metadata (floating-point gains and offsets, a pair for each band of each scene) that allow floating-point calibrated values to be recovered. While the maximum value of each band of the scene is mapped to the largest digital number (DN) of the fixed-point representation, the offsets are set equal to the minimum value of the calibrated format such that the active range of floating-point values of the scene is exactly mapped in the dynamic range of the DN representation. The offsets may be taken as equal to zero, so that the DN and the floating-point representations differ only by a scaling factor; unfortunately, they may still vary from one band to another in the same scene.

A problem that has seldom been investigated in the literature [89] is whether fusion is better accomplished in a packed fixed-point format or in a floating-point calibrated format, e.g., radiance, spectral radiance, top-of-atmosphere (TOA) reflectance, or surface reflectance. Surface reflectance is a level two (L2) product, and is generally available for global coverage systems (OLI, Sentinel-2) only when an instrument network is available for atmospheric measurements carried out by means of sun photometers or LiDAR instruments [91]. Fixed-point formats may not reflect the original calibration [89]. Packaging introduces gains and possibly offsets in order to exactly fill the wordlength without affecting the original accuracy of the data dictated by the number of bits of the onboard analog-to-digital converter (ADC). For a 12-bit ADC, the packed format is 11 bits, as the ADC is kept far from saturation and the dark signal is subtracted prior to instrumental calibration. In substance, any calibrated format can be obtained starting from the same set of DNs by means of gain–offset pairs constant for each band, at least if the sun height and atmospheric transmittance and path radiance (which are variable from one band to another) are assumed to be constant over the scene [34]. Thus, packed data are uncalibrated unless the gains are identical for all bands and the offsets are all zero. For radiometric distortions, their measure may not change with the physical or computational format, while for spectral distortions the measure cannot be independent of the format [89].

### 2.7. Meta-Analysis

A possible solution to the problem of comparative evaluations employing VO methods relies on the use of meta-analysis [92]. Meta-analysis involves taking results from primary research articles and quantitatively analyzing and synthesizing these data in an attempt to draw more robust conclusions. It has been widely used in many areas, especially medicine. Although simplified meta-analysis has previously been adopted to draw a comparative assessment of pansharpening methods based on super-resolution [48], a recently published study [49] was the first to formalized the use of meta-analysis for comparing pansharpening methods. While the focus in [49] was on direct comparison of CS, MRA, and VO by taking the numerical values of performance scores from almost one thousand publications, in the present paper our main concern is how to include the scores of methods that cannot be directly run on the test dataset in a performance comparison to determine the validation procedure of a new method.

The way in which performance scores of individual methods are made portable from one experiment to another consists of calculating a differential normalized quality/distortion index of the test method with respect to the same index attained by a highly popular standardized method having a unique definition and implementation and a reproducible method [89], i.e., when there are no adjustable parameters in the algorithm. Such a method was previously identified by [49] as GS spectral sharpening [11].

### 2.8. Benchmarking

During the past two decades, there has been consistent effort towards standardizing procedures for comparative evaluation of pansharpening methods [39,93]. However, these studies relate to contests; thus, their main concerns were with the experimental setup, visual evaluations, and choice of the most suitable quality/distortion indexes, not the benchmarks, i.e., the methods to be comparatively assessed by the test method.

The authors of the present study have participated in a project specifically targeted towards the benchmarking of pansharpening methods [9]. The warm welcome and ever-increasing popularity of this earlier work have motivated its present development in a direction that we previously left uncovered, namely, how to compare methods for which downloadable code is not available and/or that require extensive parametric optimizations from users in order to produce the top performance reported in the respective articles as a result of optimizations performed by the original authors. Due to this crucial issue, ref. [9] did not consider pansharpening methods based on VO, only traditional CS, MRA, and hybrid methods. A subsequent effort extended their comparisons to also encompass some popular VO and CNN methods [94].

As a matter of fact, VO methods may not create reproducible results; indeed, the performance attainable by such methods on a given dataset depends on the ability and willingness of the user as well as changes in the dataset itself (especially the landscape, but also the number and type of bands of the instrument). In certain cases, such as [43], the computational power necessary for processing is not affordable with standard hardware equipment. Based on these premises, even if VO methods can be freely downloaded and are equipped with their default running parameters, they are unlikely to attain performance on a new dataset that reflects the top performance these methods achieve in their respective publications. This is less so for CNN methods, where the training step can be performed offline on training datasets obtained with different instruments. Obviously, we would expect better performance if training were performed not only on the different sensors but also on different landscapes, i.e., one training for urban areas, another for rural areas, another for forests and wild areas, etc. However, a scene may contain different landscapes, and optimization is carried out based only on the utilized instrument.

The proposed protocol is summarized by the following recommendations:

1. Choose at least two different datasets, not two parts of the same image, coming from two different instruments; at least one should have a 4:1 MS-to-Pan scale ratio. A different number of bands between the two datasets is also desirable.
2. Establish the experimental setup, e.g., [9]. The setup concerns assessment (at reduced resolution or at both reduced and full resolution) of spatial interpolation, as well as spatial degradation when applicable; see Section 3 for an example.
3. Choose performance indexes that are obviously different for reduced resolution and full resolution. The performance indexes should be fairly independent of one another, specific for pansharpening, exhibit good discrimination capability, and be reasonably in-trend. It is important not to use too many indexes in order to avoid confusion. In particular, low-confidence indexes that have never been validated for pansharpening evaluations should be avoided, e.g., entropy, mutual information, average gradients, etc., as they might compromise the success of the comparative assessment.
4. Whenever possible, use a standard implementation of CS, MRA, and hybrid methods such as those provided in [9], in which a few algorithms stand out for performance and efficiency. Comparisons with up-to-date top-performing methods, though not very efficient in terms of the performance–cost tradeoff, should be performed through meta-analysis, as we demonstrate in Section 3.

## 3. Experimental Results

Experiments were carried out on real datasets acquired by five different satellite instruments over different landscapes, as reported in Table 1.

### 3.1. Benchmarks

The benchmarks consisted of twelve CS/MRA/hybrid methods plus three popular VO methods based on either modeling or learning taken from the MS Pansharpening Toolbox in [9]. The three VO methods were implementations of the authors, each optimized for one satellite scanner. Two other VO methods for which the code was unavailable from their respective publications were assessed via meta-analysis. The meta-analysis was validated by means of cross-simulations of a larger number of methods, comprising the three VO methods for which code was available. The methods are:

1. MS image interpolated with a 23-taps kernel (EXP) [15].
2. Brovey transfom (BT) [1,60].
3. Gram–Schmidt (GS) spectral sharpening method [11].
4. GS with adaptive intensity (GSA) [1,59].
5. Fast fusion with hyperspherical color space (HCS) [95].
6. SFIM technique [9,64].
7. Optimized BT with haze correction (BT-H) [33].
8. Fast fusion with hyper-ellipsoidal color space (HECS) [13].
9. Generalized LP (GLP) matched to MTF with context-based decision (CBD) [1,96].
10. Fusion method with band-dependent spatial details (BDSD) injection [1,9,97].
11. Original AWLP approach proposed in [38].
12. AWLP with haze correction (AWLP-H) [58], reviewed in Equation (16).
13. GLP with MTF filters and full-scale detail injection modeling (MTF-GLP-FS) [98].
14. Sparse representation dictionary learning pansharpening (SRDLP) [46].
15. Joint sparse and low-rank pansharpening (JSLRP) [44].
16. Fusion based on sparse representation of spatial details (SR-D) [42].
17. Fusion based on total-variation (TV) optimization [41].
18. Advanced pansharpening with neural networks and fine tuning (A-PNN-FT) [55].

Each experiment consisted of simulations performed with a subset of the 18 methods and other results achieved through meta-analysis from other tests or publications.

### 3.2. Setup

The experimental setup concerned a series of issues that standardize the comparative assessment:

- Data format; we used the spectral radiance unpacked to floating-point values,
- Interpolation filters; we used 23-tap filters [15].
- RR or FR assessment; we adopted RR assessment.
- In the case of RR assessment, we specified the reduction filters using MTF-matched Gaussian filters [24] with two cascaded stages of filtering and decimation by two.

### 3.3. Fusion Simulations

**Figure 4:**
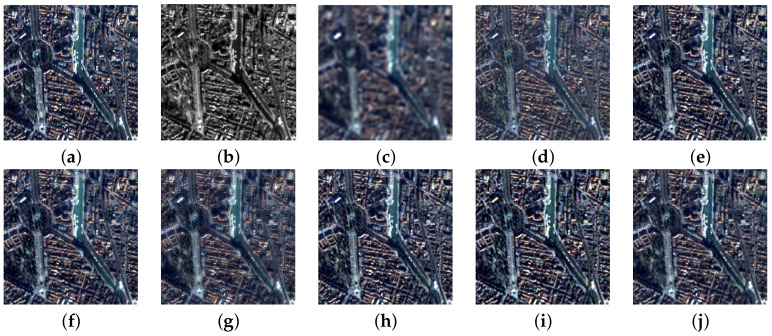
True-color 256×256 details of the IKONOS-Toulouse image at 4 m SSI: (**a**) GT; (**b**) Pan; (**c**) EXP; (**d**) GS; (**e**) GSA; (**f**) BDSD; (**g**) SFIM; (**h**) MTF-GLP-CBD; (**i**) AWLP; (**j**) AWLP-H.

**Figure 5:**
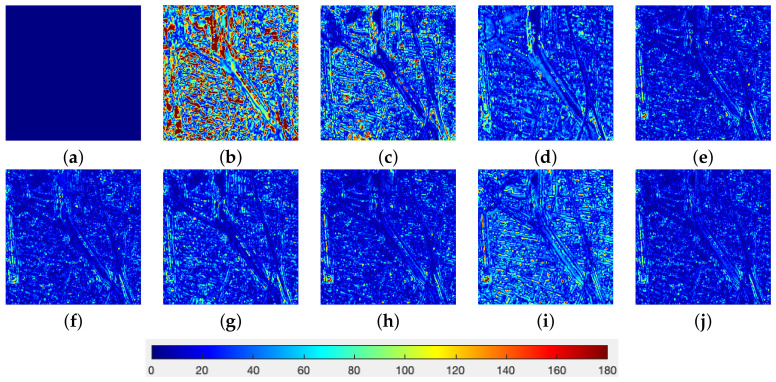
IKONOS-Toulouse image at 4 m SSI (Euclidean norm of the difference between the fused image and GT): (**a**) GT; (**b**) Pan; (**c**) EXP; (**d**) GS; (**e**) GSA; (**f**) BDSD; (**g**) SFIM; (**h**) MTF-GLP-CBD; (**i**) AWLP; (**j**) AWLP-H.

Figure 4 presents the simulations on the IKONOS-Toulouse image. The ground truth (GT) was available for RR assessments and is displayed first. The visual appearance of the results produced by the eight compared methods, including EXP, reveal a variability in performance that follows the temporal development of the methods themselves. As a common practice when there are many simulations and the details are necessarily small, the Euclidean norms of the differences between the fused bands and GT (Figure 4a) are plotted with a cold-body color bar. Figure 5 reports the maps of the $L_{2}$-norm of the error. The Pan image in Figure 4b is regarded as a fusion product, and its components are taken to be all equal to the original grayscale Pan, which causes the error in Figure 5b to blow up.

**Figure 6:**
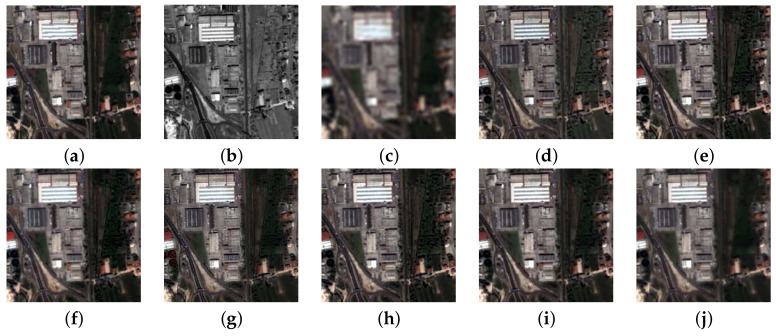
True-color 256×256 details of the QuickBird-Trento image at 3.2 m SSI: (**a**) GT; (**b**) Pan; (**c**) EXP; (**d**) GS; (**e**) GSA; (**f**) BDSD; (**g**) SFIM; (**h**) MTF-GLP-CBD; (**i**) AWLP; (**j**) AWLP-H.

Figure 6 shows the simulation results of the QuickBird-Trento image. The methods are a subset of the set of benchmarks, and are exactly the same as for the previous simulation results shown in Figure 4. When the comparison concerns a vegetated landscape, all the methods with the exception AWLP-H (and, trivially, EXP) suffer from a noticeable over-enhancement of the tree canopy, which is missing in the GT. This effect originates from Pan textures injected into the blue band [57], and is mitigated by the correction of AWLP-H.

**Figure 7:**
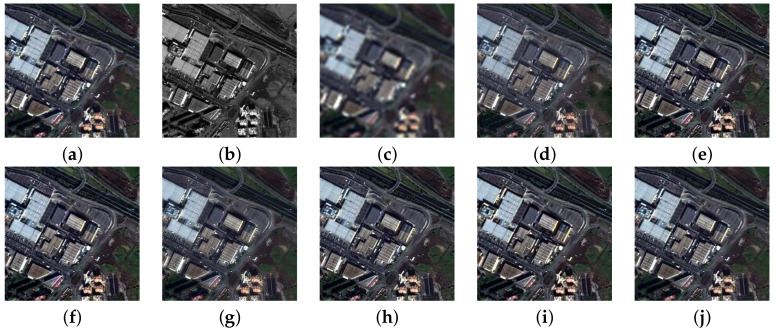
True-color 256×256 details of the WorldView-2 Rome image at 2 m SSI: (**a**) GT; (**b**) Pan; (**c**) EXP; (**d**) GS; (**e**) GSA; (**f**) BDSD; (**g**) SFIM; (**h**) MTF-GLP-CBD; (**i**) AWLP; (**j**) AWLP-H.

Figure 7 portrays the simulation results for the WorldView-2 Rome image. The methods were the same as those for the two previous simulations shown in Figure 4 and Figure 6, and the same considerations apply to the visual assessment.

Table 2 shows the quantitative assessment for the first three test images. It is clear that AWLP-H attains the best performance in all quality metrics for both datasets. It is remarkable that there is a consistent reduction in SAM value with respect to baseline AWLP, which clamps the spectral angle of the interpolated image. This consideration is strengthened by the visual analysis of Figure 4, Figure 6 and Figure 7, where spectral distortions of some fused products are noticeable (see, e.g., Figure 4d,i). On the other hand, Figure 4g shows that SFIM has good spectral accuracy but poor spatial accuracy.

### 3.4. Meta-Analysis

Next, we investigate the portability of quality/distortion values across different experiments. A few years ago, an article on a model-based VO [44] reported the performance of the up-to-date JSLRP and another popular VO-based method by the same coauthor named SRLDP [46]. Both used the WorldView-2 dataset related to the city of Sydney, Australia. This allows for a meta-analysis taking the JSLRP and SRLDP scores from [44] and using GS as a standard benchmark. Original scores (Q8, SAM and ERGAS) attained by each of the two VO methods in [44] were standardized using the respective GS scores calculated during the same experiment. For the test method (TM), either JSLRP and SRLDP in the present case or the standard GS method as recommended in [49], we defined the normalized differential score indexes (NDSI) shown below:

(18) $$\begin{array}{ccc} \Delta Q8\% & ≜ & \frac{Q8(\mathrm{TM})-Q8(\mathrm{GS})}{Q8(\mathrm{GS})}\times 100\\ \Delta \mathrm{SAM}\% & ≜ & \frac{\mathrm{SAM}(\mathrm{TM})-\mathrm{SAM}(\mathrm{GS})}{\mathrm{SAM}(\mathrm{GS})}\times 100\\ \Delta \mathrm{ERGAS}\% & ≜ & \frac{\mathrm{ERGAS}(\mathrm{TM})-\mathrm{ERGAS}(\mathrm{GS})}{\mathrm{ERGAS}(\mathrm{GS})}\times 100. \end{array}$$

The NDSIs of JSLRP and SRLDP were calculated from the values in [44] as follows: (10.17−22.87−8.80) and (12.32−29.11−25.47) for (Q8 SAM ERGAS), respectively. These were translated into the three experiments and denormalized by the respective values of the GS pivot algorithm to yield the last two rows of Table 3, which are relative to a different experiment on a different dataset of the same instrument. Then, Equation (18) was inverted to yield the inferred Q8, SAM, and ERGAS reported in Table 3 (in the entries SRLDP and JSLRP). The results of our WorldView-2 experiment enhanced by meta-analysis of [44] reveal that AWLP-H is superior to all methods in the previously considered Pansharpening Toolbox [9], as well as SRLDP [46], but not JSLRP [44], which is an up-to-date method from the VO class. The increase in JSLRP over AWLP-H in terms of Q8 is equal to 0.015, or approximately 1.8%. Notably, AWLP-H yields reproducible results without manual adjustments, while JSLRP is a VO method that requires massive computation and extensive parametric adjustments from the user.

**Figure 8:**
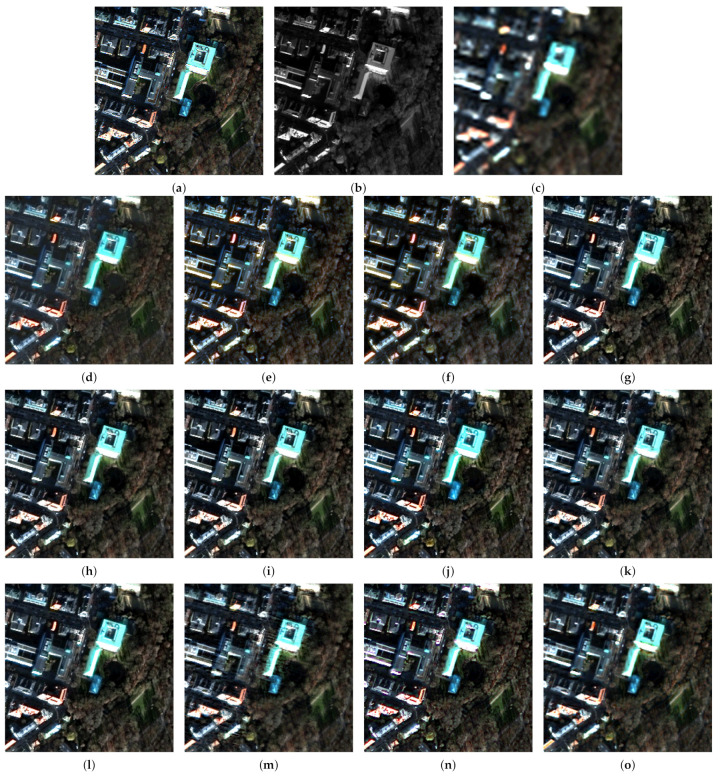
Fusion results for the Munich dataset at reduced resolution using a true-color representation: (**a**) Reference; (**b**) Pan image; (**c**) expanded; (**d**) GS; (**e**) BT; (**f**) HCS; (**g**) GSA; (**h**) BT-H; (**i**) HECS; (**j**) BDSD; (**k**) AWLP-H; (**l**) MTF-GLP-FS; (**m**) SR-D; (**n**) TV; (**o**) A-PNN-FT.

**Figure 9:**
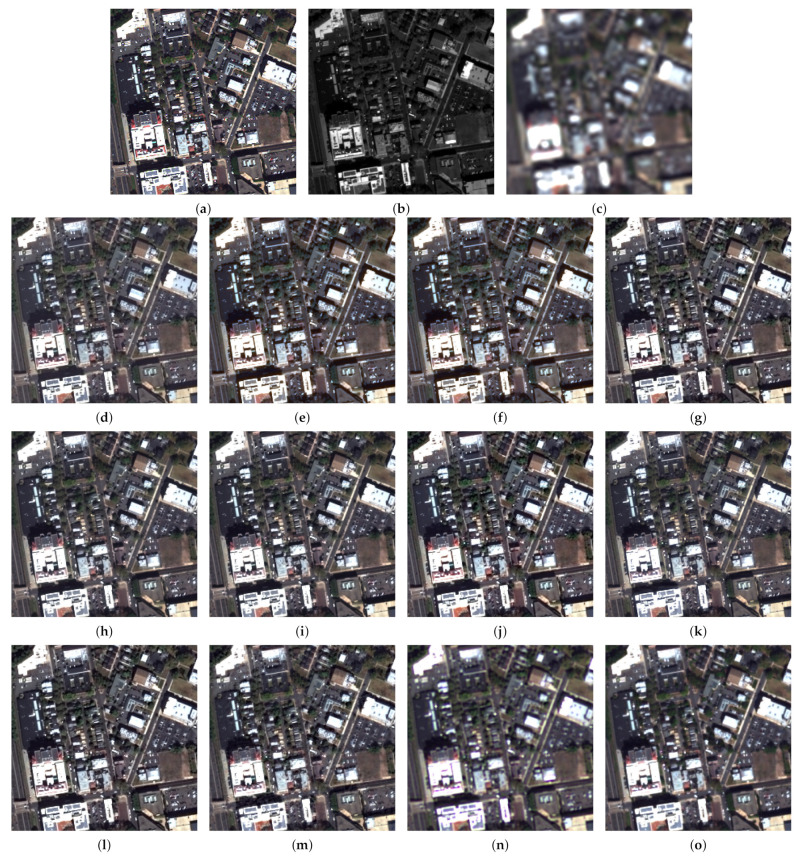
Fusion results for the Trenton dataset at reduced resolution using a true-color representation: (**a**) Reference; (**b**) Pan image; (**c**) expanded; (**d**) GS; (**e**) BT; (**f**) HCS; (**g**) GSA; (**h**) BT-H; (**i**) HECS; (**j**) BDSD; (**k**) AWLP-H; (**l**) MTF-GLP-FS; (**m**) SR-D; (**n**) TV; (**o**) A-PNN-FT.

Next, we investigated the portability of meta-analysis across experiments. Table 4 reports the results for the WorldView-3 Munich and GeoEye-1 Trenton datasets with an enlarged number of methods, including three VO algorithms contained in the Pansharpening Toolbox. The visual results of the fusion are shown in Figure 8 and Figure 9.

We then changed the pivot and tried to calculate the values of the three indexes via meta-analysis (Munich from Trenton and Trenton from Munich). In this way, it is possible to quantify the error of the meta-analysis to find the best pivot and most portable index. We exhaustively tested all pivots, finding that the choice of GS in [49] was dictated by the requirement of a standard implementation. In the present study, the same codes were run for the two datasets. We found that the best performing methods with a high degree of reproducibility increased the accuracy of the meta-analysis when used in Equation (18) in place of GS. Table 5 contains the results of the crossed meta-analysis between Munich and Trenton calculated using AWLP-H as a pivot. The differences with respect to the true values in Table 4 are shown in Table 6. The mean absolute value of the errors normalized to the mean of true values (NMAE%) was calculated for each index and each dataset, and is reported in the last row of Table 6. What immediately stands out is that Q$2^{n}$ is by far the most portable index, thanks to a structure that balances spectral and spatial distortions. This means that the values of Q$2^{n}$ are highly portable and that AWLP-H is adequate as pivot, as its results vary steady with the landscape and instrument, mostly thanks to the haze correction. Incidentally, the values of NMAE% for Q$2^{n}$ and different pivots are as follows:

- GS: 7.12% Munich, 7.67% Trenton.
- BT: 4.69% Munich, 4.90% Trenton.
- AWLP-H: 3.19% Munich, 3.18% Trenton.
- HECS: 3.14% Munich, 3.13% Trenton.

In fact, HECS [13], which features haze correction, is the most suitable method for pivoting. Not surprisingly, HECS performs the best in Table 4. This supports our conjecture that the pivot should be reproducible, highly performing, and most importantly widespread. The meta-analysis, however, can be performed with different pivots. Unfortunately, the results for SRDLP and JSRLP in Table 3 cannot be inferred with a pivot other than GS, as AWLP-H and HECS were published after [44].

## 4. Discussion

This example of benchmarking raises a number of issues. Fair benchmarking is crucial for the validation of new methods. If any elements are unfair, the significance of the comparative evaluation can be compromised. The first issue is that the benchmarks should be reproducible and non-parametric. Our experiment including a meta-analysis revealed that despite their extensive parametric optimizations, which often imply massive computational requirements, pansharpening methods based on sparse representation may be unrewarding compared to second-generation methods based on simplified physical models of instruments (e.g., MTF) and radiative transfer through atmosphere (e.g., dehazing), which are often simpler and faster. Unfortunately, meta-analysis does not describe the behavior of fusion methods, in the sense that sparse methods can produce mathematically optimized results by visually unlikely enhancements (in other words, localized visual artifacts accompanied by high global quality scores, e.g., in the presence of local misalignment) [83]. Visual analysis is also important for detecting gross errors due to improper setups and anomalous data sets. In fact, statistical indexes yield average values; local defects (e.g., MS-to-Pan shifts and aliasing of MS) produce localized errors to a small extent on average, although these may be visually unpleasant or even annoying.

Further insight into our meta-analysis reveals that $Q2^{n}$ is highly portable across different experiments, with SAM and ERGAS much less so. However, the use of more than one index can expose inconsistencies in data sets and in the implementation both of fusion and of assessment. The real importance here lies in the availability of widespread standard implementations of benchmarks. The example of the TV algorithm is illuminating; the same code produces results that are excellent in the Munich data set (Figure 8n) and mediocre in the Trenton data set (Figure 9n), probably because its parameters were optimized in the former. This anomaly is reflected in the scores in Table 4. In Table 5, where Trenton is inferred from Munich by meta-analysis, the scores are consistently better; however, the scores of Munich are poor, being inferred from Trenton.

## 5. Conclusions

The focus of this article is on the benchmarking of pansharpening algorithms. A series of recommendations are presented and discussed with the aim of producing fair comparative valuations of methods. Meta-analysis is introduced and the optimal choice of the method that serves as a pivot is investigated. Through meta-analysis, a large number of methods can be compared simply with the aid of a spreadsheet.

A benchmarking protocol for MS pansharpening has been proposed and validated on five different datasets. The choice of methods for comparative evaluation should follow two main guidelines: wherever the methods provide reproducible results varying with the dataset, reliable implementations, e.g., those of [9], can be run on the test dataset; alternatively, when using a benchmark that requires extensive optimization varying with the dataset, its performance scores should instead be inferred from those of the original publication by applying a meta-analysis rather than trying to optimize the results of the method on the test dataset. Meta-analysis ensures the portability of performance scores through experiments carried out on different datasets.

Our application of the proposed benchmarking protocol placed AWLP-H first among the algorithms compared in the first three tests used for benchmarking. In the Rome test, AWLP-H is second, with a deviation of 1.8%, when two popular and highly performing methods based on variational optimization [44,46] were included. The latter comparison is feasible by resorting to meta-analysis. A recent effort of the authors, HECS [13], exhibits favorable characteristics in terms of performance, reproducibility, and suitability for pivoting. In fact, both AWLP-H and HECS are good candidates to serve as the pivot in the meta-analysis process. The implementation of HECS will soon be available in the Pansharpening Toolbox. Eventually, the family of $Q2^{n}$ quality indexes is expected to provide portable meta-analysis across different tests.

## Figures and Tables

**Table 1:** Datasets used in this study and related information. The bands are as follows: coastal (C), blue (B), green (G), yellow (Y), red (R), red edge (RE), near-infrared (NIR), and outermost NIR (NIR2); SSI stands for spatial sampling interval, and is relative to the geocoded product.

Dataset	Satellite	Location & Date	SSI [m]	Spectral Bands	Scene Size	Format
1	IKONOS	Toulouse, France	1.0	Panchromatic	2048 × 2048	TOA Spectral Radiance
		15 May 2000	4.0	B, G, R, NIR	512 × 512	from 11-b DNs
2	QuickBird	Trento, Italy	0.7	Panchromatic	1024 × 1024	TOA Spectral Radiance
		October 2005	2.8	B, G, R, NIR	256 × 256	from 11-b DNs
3	WorldView-2	Rome, Italy	0.5	Panchromatic	1200 × 1200	TOA Spectral Radiance
		18 September 2013	2.0	B, G, R, NIR	300 × 300	from 11-b DNs
				C, Y, RE, NIR2	300 × 300	
4	WorldView-3	Munich, Germany	0.4	Panchromatic	2048 × 2048	TOA Spectral Radiance
		10 January 2020	1.6	B, G, R, NIR	512 × 512	from 11-b DNs
				C, Y, RE, NIR2	512 × 512	
5	GeoEye-1	Trenton, NJ, USA	0.5	Panchromatic	2048 × 2048	TOA Spectral Radiance
		27 September 2019	2.0	B, G, R, NIR	512 × 512	from 11-b DNs

**Table 2:** Fusion comparison at RR for the IKONOS-Toulouse, QuickBird-Trento, and WordlView-2 Rome datasets. GT indicates the reference ground truth. The best values are shown in bold and the second-best values are in italic. All algorithms were run in spectral radiance format.

Dataset	Toulouse			Trento			Rome		
	Q4	SAM	ERGAS	Q4	SAM	ERGAS	Q8	SAM	ERGAS
**GT**	1	0	0	1	0	0	1	0	0
**EXP**	0.519	4.840	5.879	0.785	*3.343*	3.645	0.715	4.982	5.479
**GS**	0.808	4.260	4.191	0.766	5.110	3.923	0.830	4.907	4.052
**GSA**	0.932	3.021	2.586	0.833	4.193	3.316	0.890	4.157	3.398
**BDSD**	*0.931*	*2.800*	*2.467*	*0.862*	3.663	2.979	0.875	4.973	3.866
**SFIM**	0.866	3.615	3.519	0.841	3.835	5.951	0.891	4.146	3.449
**CBD**	0.933	3.016	2.566	0.849	4.040	3.059	0.893	4.159	3.354
**AWLP**	0.897	4.840	3.262	0.861	*3.343*	*2.937*	0.799	4.982	3.563
**AWLP-H**	**0.936**	**2.756**	**2.433**	**0.889**	**3.093**	**2.637**	**0.917**	**3.605**	**3.114**

**Table 3:** Fusion comparison at RR for the IKONOS-Toulouse, QuickBird-Trento, and WordlView-2 Rome datasets. GT indicates the reference ground truth. The best values are shown in bold and the second-best values are in italic. All algorithms were run in spectral radiance format. The results of SRDLP and JSRLP were inferred from [44]. For the two four-band datasets, the Q8 meta-analysis results were treated as Q4 and placed in the corresponding columns.

Dataset	Toulouse			Trento			Rome		
	**Q4**	**SAM**	**ERGAS**	**Q4**	**SAM**	**ERGAS**	**Q8**	**SAM**	**ERGAS**
**GT**	1	0	0	1	0	0	1	0	0
**EXP**	0.519	4.840	5.879	0.785	*3.343*	3.645	0.715	4.982	5.479
**GS**	0.808	4.260	4.191	0.766	5.110	3.923	0.830	4.907	4.052
**GSA**	0.932	3.021	2.586	0.833	4.193	3.316	0.890	4.157	3.398
**BDSD**	*0.931*	*2.800*	*2.467*	*0.862*	3.663	2.979	0.875	4.973	3.866
**SFIM**	0.866	3.615	3.519	0.841	3.835	5.951	0.891	4.146	3.449
**CBD**	0.933	3.016	2.566	0.849	4.040	3.059	0.893	4.159	3.354
**AWLP**	0.897	4.840	3.262	0.861	*3.343*	2.937	0.799	4.982	3.563
**AWLP-H**	**0.936**	**2.756**	**2.433**	**0.889**	**3.093**	**2.637**	*0.917*	*3.605*	*3.114*
**SRDLP**	0.890	3.286	3.822	0.844	3.941	3.578	0.914	3.785	3.696
**JSRLP**	0.908	3.020	3.124	0.860	3.622	*2.924*	**0.932**	**3.479**	**3.020**

**Table 4:** Fusion comparison at RR for the WorldView-3 Munich and GeoEye-1 Trenton datasets. GT indicates the reference ground truth. The best values are shown in bold and the second-best values are underlined. All algorithms were run in spectral radiance format except A-PNN-FT, for which the results were converted to spectral radiance before assessment due to its requiring the same DN format used for training.

Dataset	Munich			Trenton		
	**Q8**	**SAM**	**ERGAS**	**Q4**	**SAM**	**ERGAS**
**GT**	1.0000	0.0000	0.0000	1.0000	0.0000	0.0000
**EXP**	0.6311	4.7548	10.8511	0.5826	6.6167	10.2034
**BT**	0.8803	4.7548	5.5754	0.9000	6.6167	5.3655
**GS**	0.8028	4.2535	6.9518	0.8461	6.2997	6.6388
**HCS**	0.8906	4.7548	6.1731	0.8969	6.6167	5.4681
**BT-H**	0.9236	2.9309	4.2466	0.9025	4.9937	4.9978
**GSA**	0.9204	3.2007	4.4250	0.8985	6.0420	5.2664
**HECS**	**0.9287**	**2.9078**	**4.1268**	**0.9066**	4.9565	**4.9609**
**BDSD**	0.9245	3.2388	4.1748	0.9054	6.0254	5.1267
**AWLP-H**	0.9154	2.9794	4.3915	0.8928	5.2913	5.2182
**MTF-GLP-FS**	0.9200	3.1876	4.4465	0.9030	6.0093	5.1501
**SR-D**	0.8936	3.4386	5.3399	0.8915	5.4449	5.3810
**TV**	0.9164	3.4225	4.6557	0.7693	6.1318	7.7066
**A-PNN-FT**	0.8747	3.6465	5.8899	0.8857	**4.3841**	5.4262

**Table 5:** Values of quality/distortion indexes for WorldView-3 Munich and GeoEye-1 Trenton calculated via the crossed meta-analysis from Table 4. AWLP-H was used as pivot. Q8 and Q4 are interchangeable; Q4 of Trenton was inferred from Q8 of Munich and vice versa.

Dataset	Munich			Trenton		
	**Q8**	**SAM**	**ERGAS**	**Q4**	**SAM**	**ERGAS**
**EXP**	0.5973	3.7257	8.5869	0.6155	8.4443	12.8938
**BT**	0.9228	3.7257	4.5155	0.8586	8.4443	6.625
**GS**	0.8675	3.5472	5.587	0.783	7.5541	8.2605
**HCS**	0.9196	3.7257	4.6018	0.8686	8.4443	7.3352
**BT-H**	0.9253	2.8118	4.206	0.9008	5.2052	5.046
**GSA**	0.9212	3.4021	4.4321	0.8977	5.6843	5.258
**HECS**	0.9295	2.7909	4.175	0.9058	5.1641	4.9037
**BDSD**	0.9283	3.3928	4.3145	0.9017	5.752	4.9607
**AWLP-H**	0.9154	2.9794	4.3915	0.8928	5.2913	5.2182
**MTF-GLP-FS**	0.9259	3.3837	4.3342	0.8973	5.6611	5.2836
**SR-D**	0.9141	3.0659	4.5285	0.8715	6.1068	6.3451
**TV**	0.7888	3.4527	6.4857	0.8938	6.0782	5.5321
**A-PNN-FT**	0.9081	2.4686	4.5665	0.8531	6.476	6.9987

**Table 6:** Inferred scores of Table 5 with AWLP-H used as pivot, decremented by the true scores in Table 4. The last row reports the NMAE% for each index and each test image.

Dataset	Munich			Trenton		
	**Q8**	**SAM**	**ERGAS**	**Q4**	**SAM**	**ERGAS**
**EXP**	−0.0338	−1.0291	−2.2642	0.0329	1.8276	2.6904
**BT**	0.0425	−1.0291	−1.0599	−0.0414	1.8276	1.2595
**GS**	0.0647	−0.7063	−1.3648	−0.0631	1.2544	1.6217
**HCS**	0.0290	−1.0291	−1.5713	−0.0283	1.8276	1.8671
**BT-H**	0.0017	−0.1191	−0.0406	−0.0017	0.2115	0.0482
**GSA**	0.0008	0.2014	0.0071	−0.0008	−0.3577	−0.0084
**HECS**	0.0008	−0.1169	0.0482	−0.0008	0.2076	−0.0572
**BDSD**	0.0038	0.1540	0.1397	−0.0037	−0.2734	−0.1660
**AWLP-H**	-	-	-	-	-	-
**MTF-GLP-FS**	0.0059	0.1961	−0.1123	−0.0057	−0.3482	0.1335
**SR-D**	0.0205	−0.3727	−0.8114	−0.0200	0.6619	0.9641
**TV**	−0.1276	0.0302	1.8300	0.1245	−0.0536	−2.1745
**A-PNN-FT**	0.0334	−1.1779	−1.3234	−0.0326	2.0919	1.5725
**NMAE %**	**3.19**	12.98	14.84	**3.18**	14.51	16.33

## Data Availability

The image datasets analyzed in this study can be found at: https://resources.maxar.com/product-samples/pansharpening-benchmark-dataset/ (subject to authorization by Maxar, accessed on 15 October 2024) and https://eoiam-idp.eo.esa.int/ (subject to authorization by ESA, accessed on 15 October 2024).

## Abbreviations

The following abbreviations are used in this manuscript:

ADC	Analog-to-Digital Converter
BDSD	Band-Dependent Spatial Detail
CS	Component Substitution
EHR	Extremely High Resolution
EO	Earth Observation
ERGAS	Erreur Relative Globale Adimensionnelle de Synthèse
FR	Full Resolution
GLP	Generalized Laplacian Pyramid
GS	Gram–Schmidt
HCS	Hyperspherical Color Space
HECS	Hyper-Ellipsoidal Color Space
HPM	High-Pass Modulation
IHS	Intensity–Hue–Saturation
LiDAR	Light Detection And Ranging
LP	Laplacian Pyramid
MMSE	Minimum Mean Square Error
MRA	Multi-Resolution Analysis
MS	Multi-Spectral
MSE	Mean Square Error
MTF	Modulation Transfer Function
NRMSE	Normalized Root Mean Square Error
NIR	Near Infra-Red
OLI	Operational Land Imager
PCA	Principal Component Analysis
QNR	Quality with No Reference
RMSE	Root Mean Square Error
RR	Reduced Resolution
RS	Remote Sensing
SAM	Spectral Angle Mapper
SAR	Synthetic Aperture Radar
SNR	Signal-to-Noise Ratio
SPOT	Satellite Pour l’Observation de la Terre
SSI	Spatial Sampling Interval
SWIR	Short-Wave Infra-Red
TIR	Thermal Infra-Red
UIQI	Universal Image Quality Index
VHR	Very High Resolution
VNIR	Visible Near-Infra-Red
